# Cognitive Impairment in Schizophrenia Across Different Clinical Manifestations and Its Association With Symptom Severity and Functional Status

**DOI:** 10.1002/brb3.71734

**Published:** 2026-09-07

**Authors:** Yao Chen, Kanghua Zhang, Yu Mu, Xuezhen Jia

**Affiliations:** ^1^ Department of Psychosomatic Medicine, Nanjing Pukou People's Hospital Liangjiang Hospital Southeast University Nanjing Jiangsu People's Republic of China; ^2^ Department of Geriatrics, Changning District Mental Health Center The Affiliated Mental Health Center of East China Normal University Shanghai People's Republic of China; ^3^ Child and Adolescent Ward, Department of Psychiatry Jinhua Second Hospital Jinhua Zhejiang People's Republic of China

**Keywords:** cognitive function, negative symptoms, positive symptoms, schizophrenia, social function

## Abstract

**Objective:**

To explore the cognitive impairment in patients with schizophrenia of different clinical manifestations and its relationship with symptom severity and functional status.

**Methods:**

The clinical data of 160 patients with schizophrenia who were treated in our hospital from April 2019 to December 2025 were selected for the study. The Positive and Negative Syndrome Scale (PANSS) scores were used to classify patients into the positive group (*n* = 93) and the negative group (*n* = 67). The recorded cognitive scores, PANSS scores, and Personal and Social Performance (PSP) scores were compared. Distributional assumptions were examined before analysis; Welch's *t*‐test and Spearman rank correlation were used, and Hedges' *g* with 95% confidence intervals was reported for significant between‐group comparisons.

**Results:**

The positive group had higher PANSS positive symptom and PSP scores but lower PANSS negative symptom scores than the negative group (all *p* < 0.001). The positive group also showed higher recorded cognitive total and domain scores (all *p* < 0.001). Spearman analysis showed that PANSS negative symptom scores were inversely correlated with the recorded cognitive total score and PSP score (rho = −0.350 and −0.464, respectively), whereas PANSS positive symptom scores were positively correlated with these outcomes (rho = 0.652 and 0.781, respectively; all *p* < 0.001). The recorded cognitive total score was positively correlated with PSP score (rho = 0.655, *p* < 0.001).

**Conclusion:**

Cognitive performance differs across clinical symptom profiles in schizophrenia. Patients with predominant negative symptoms showed poorer cognitive performance and social functioning than patients with predominant positive symptoms. These findings support assessment of symptom profile, cognition, and functioning together, without implying causality.

## Graphical abstract

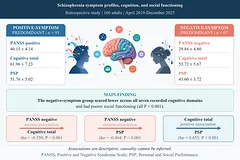

## 1 Introduction

Schizophrenia is a common psychiatric disorder characterized by highly heterogeneous clinical manifestations. The disorder not only affects patients’ mental health but also causes substantial impairment in cognitive and social functioning (Ganeshalingam et al. 2025). Cognitive impairment is considered one of the core features of schizophrenia, affecting speed of information processing, attention, memory, and executive function, and may persist throughout the entire course of the disease (Ward et al. 2025). However, the underlying mechanisms responsible for cognitive dysfunction in schizophrenia have not yet been fully elucidated. Current evidence suggests that it may be associated with factors such as imbalance in neurotransmitter systems, abnormalities in brain network connectivity, and immune‐inflammatory responses.

Based on clinical symptom characteristics, schizophrenia can generally be classified into a positive symptom‐predominant type and a negative symptom‐predominant type (J. Zhang, Tusuzian, et al., 2025). Positive symptoms mainly reflect excessive or distorted mental activity, such as hallucinations, delusions, and disorganized thinking. In contrast, negative symptoms are characterized by a reduction or loss of normal mental functions, including affective blunting, alogia (poverty of speech), and avolition (reduced motivation). Previous research has reported that cognitive impairment in patients with schizophrenia is associated with both positive and negative symptoms, with negative symptoms exerting a more pronounced impact on cognitive function (Du et al. 2025). One possible explanation is that patients with predominant negative symptoms are more difficult to identify and diagnose during the early stages of the disease, resulting in a longer duration of untreated illness compared with patients whose symptoms are predominantly positive. In addition, such patients often lack motivation to actively acquire information, engage in reflective thinking, or participate in social interactions. These factors may impair daily functioning, such as employment and social engagement, and further exacerbate cognitive impairment. Conversely, cognitive impairment may limit patients’ ability to participate in effective social interactions, creating a vicious cycle that further restricts recovery of social functioning. Although positive symptoms, such as auditory hallucinations and delusions, may interfere with thinking processes to some extent, they are more often manifested as abnormalities in the content of thought rather than fundamental deficits in thinking ability.

Systematic reviews and meta‐analyses have shown that negative symptoms are consistently associated with poorer performance across several neurocognitive domains, whereas associations involving positive symptoms are generally weaker and less consistent. Meta‐analytic evidence also indicates that both neurocognition and social cognition are related to functional outcomes (Au‐Yeung et al. 2023; Fett et al. 2011). These higher level findings provide the theoretical basis for jointly evaluating symptom dimensions, cognitive performance, and real‐world functioning in the same clinical sample.

Based on these considerations, we hypothesized that patients with schizophrenia characterized by predominant negative symptoms may exhibit more severe impairment in both cognitive and social functioning. To further clarify these associations, the present study retrospectively analyzed the clinical data of 160 patients with schizophrenia. We aim to investigate the associations of positive and negative symptoms with cognitive as well as social functioning, thereby providing evidence to inform clinical practice.

## 2 Materials and Methods

### 2.1 Clinical Data

The sample size was calculated using the following formula:$n=\frac{Z_{1-a/2}^{2}\times P\left(1-P\right)}{E^{2}}$, where *Z*_1‐a_ = 1.96 is the critical value for the confidence level; *p* is the expected proportion, and *E* is the allowable error. According to Dai et al. (2017), their study included 80 patients with schizophrenia, 36 exhibited negative symptoms (*p*_0_ = 0.4500). Clinically, among 27 patients with schizophrenia, 10 were estimated to have negative symptoms (*p*_1_ = 0.3704), with the probability difference set as *E* = 0.0796. Accordingly, the calculated sample size was *n* = 150. Thus, 150 represented the minimum required sample size, whereas the final analysis included 160 eligible patients.

This study retrospectively analyzed clinical data from 160 patients with schizophrenia treated at our hospital between April 2019 and December 2025. Based on the Positive and Negative Syndrome Scale (PANSS), patients were classified into positive (*n* = 93) and negative (*n* = 67) groups.

Inclusion criteria were as follows: (1) Diagnosis of schizophrenia according to the Diagnostic and Statistical Manual of Mental Disorders, Fifth Edition (DSM‐5) and the International Classification of Diseases, 10th Revision (ICD‐10) for Mental and Behavioral Disorders (Fan 1993), confirmed clinically, (2) age ≥ 18 years, (3) PANSS total score ≥ 60 points, (4) no prior use of antipsychotic medications or cumulative antipsychotic treatment duration < 4 weeks before enrollment, and (5) complete clinical records.

Exclusion criteria included (1) comorbid Axis I psychiatric disorders; (2) pregnancy or lactation; (3) history of electroconvulsive therapy (ECT) or transcranial magnetic stimulation (TMS); and (4) evident suicidal or homicidal behavior.

Patient classification criteria were established according to PANSS scores (Wang et al. 2015). Those with ≥ 3 items scored ≥ 4 on the PANSS positive symptom subscale, but fewer than 3 items scored ≥ 4 on the negative subscale, were assigned to the positive group. Patients with ≥ 3 items scored ≥ 4 on the negative symptom subscale, but fewer than 3 items scored ≥ 4 on the positive subscale, were assigned to the negative group.

### 2.2 Methods

Severity of Schizophrenia symptom was assessed using PANSS (Si et al. 2004), which includes three dimensions: positive symptoms (seven items), negative symptoms (seven items), and general psychopathology symptoms (16 items). Each item is rated on a 7‐point scale (1–7), with higher scores indicating more severe symptoms.

Cognitive function was assessed using the MATRICS Consensus Cognitive Battery (MCCB) (Lystad et al. 2014). The official MCCB contains 10 tests covering seven cognitive domains; however, the archived dataset contained seven available domain‐aligned measures: Symbol Coding, Continuous Performance Test‐Identical Pairs (CPT‐IP), Stroop Color‐Word Test, Hopkins Verbal Learning Test‐Revised (HVLT‐R), Brief Visuospatial Memory Test‐Revised (BVMT‐R), Mazes subtest, and Mayer‐Salovey‐Caruso Emotional Intelligence Test (MSCEIT). Accordingly, the analyses used the recorded task scores, seven recorded domain scores, and the recorded total score in the source dataset. Because not all official MCCB subtests were available, these outcomes should be interpreted as a selected MCCB‐aligned cognitive assessment rather than a complete official MCCB administration. Higher scores indicate better cognitive performance.

Social functioning was assessed using the Personal and Social Performance (PSP) Scale (White et al. 2016), a clinician‐rated scale covering four domains: socially useful activities, personal and social relationships, self‐care, and disturbing and aggressive behavior. The clinician assigns a global score ranging from 1 to 100, with higher scores indicating better personal and social functioning.

All assessments were conducted independently by two attending psychiatrists.

### 2.3 Statistical Analysis

Data were analyzed using SPSS version 22.0 (IBM Corp., Armonk, NY, USA). Continuous variables were expressed as mean ± standard deviation, and categorical variables were expressed as *n* (%). Categorical variables were compared using the chi‐square (*χ* ^2^) test. Distributional assumptions were evaluated using the Shapiro–Wilk test, and homogeneity of variance was examined using Levene's test. Most symptom and cognitive variables showed departures from normality, and unequal variances were observed for several between‐group comparisons. Therefore, Welch's *t*‐test was used for continuous between‐group comparisons. Mann–Whitney *U* tests were additionally performed as sensitivity analyses and produced the same pattern of statistical significance. Because the distributional assumptions for Pearson correlation were not consistently met, Spearman rank correlation was used to evaluate associations among PANSS positive and negative symptom scores, the recorded cognitive total score, and PSP score. Hedges' *g* with 95% confidence intervals was reported as the effect‐size measure for continuous between‐group comparisons, while Spearman's rho was interpreted as the effect‐size measure for correlation analyses. All tests were two‐sided, and statistical significance was set at *p* < 0.05.

## 3 Results

### 3.1 Baseline Characteristics

There were no significant differences in baseline characteristics between the two groups (*p* > 0.05) (Table 1).

**TABLE 1:** Comparison of baseline characteristics between the two groups.

Groups	Sex, *n* (%)		Age (years)	BMI (kg/m^2^)	Education level, *n* (%)			Family history,*n* (%)	Smoking history, *n* (%)	Drinking history, *n* (%)
	Male	Female			Middle school and below	Senior high school	Technical degree and above			
Positive group (*n* = 93)	48 (51.61)	45 (48.39)	39.02 ± 5.09	23.98 ± 2.80	32 (34.41)	26 (27.96)	35 (37.63)	18 (19.35)	25 (26.88)	11 (11.83)
Negative group (*n* = 67)	34 (50.75)	33 (49.25)	39.10 ± 4.90	24.08 ± 2.72	25 (37.31)	28 (41.79)	14 (20.9)	7 (10.45)	17 (25.37)	6 (8.96)
*χ* ^2^/Welch *t*	0.012		−0.104	−0.211	5.864			2.344	0.046	0.338
*P*	0.914		0.917	0.833	0.053			0.126	0.831	0.561

### 3.2 PANSS and PSP Scores

The negative‐symptom group had lower PANSS positive symptom scores and PSP scores, but higher PANSS negative symptom scores compared with the positive‐symptom group (*p* < 0.05). No significant difference was observed in PANSS general psychopathology scores between the groups (*p* > 0.05) (Table 2).

**TABLE 2:** Comparison of PANSS and PSP scores between the two groups (points).

Groups	PANSS scores			PSP scores
	Positive symptom scores	Negative symptom scores	General psychopathology symptom scores	
Positive group (*n* = 93)	40.15 ± 4.14	21.77 ± 3.23	38.66 ± 2.92	51.76 ± 5.02
Negative group (*n* = 67)	25.70 ± 2.95	29.84 ± 4.60	38.69 ± 3.08	43.60 ± 3.72
Welch *t*	25.748	−12.316	−0.063	11.827
*p*	< 0.001	< 0.001	0.949	< 0.001
Hedges' *g* (95% CI)	3.893 (3.361 to 4.425)	−2.076 (−2.465 to −1.688)	−0.010 (−0.324 to 0.304)	1.798 (1.427 to 2.170)

### 3.3 Cognitive Function

The negative group showed significantly lower recorded cognitive total and domain scores, including speed of information processing, attention/vigilance, working memory, verbal learning, visual learning, reasoning and problem solving, and social cognition (all *p* < 0.001) (Table 3). Recorded task scores were also consistently lower in the negative group than in the positive group (all *p* < 0.001) (Table 4). Sensitivity analyses using the Mann–Whitney *U* test yielded the same pattern of statistical significance as the primary Welch's *t*‐test analyses.

**TABLE 3:** Comparison of recorded domain and cognitive total scores between the two groups (points).

Groups	Speed of information processing	Attention/vigilance	Working memory	Verbal learning	Visual learning	Reasoning and problem solving	Social cognition	Total score
Positive group (*n* = 93)	52.53 ± 10.07	52.70 ± 11.44	52.70 ± 10.37	53.42 ± 10.55	52.31 ± 10.00	55.74 ± 9.47	52.38 ± 9.53	61.96 ± 7.23
Negative group (*n* = 67)	46.28 ± 8.46	46.91 ± 6.37	46.39 ± 8.27	46.37 ± 7.54	46.78 ± 9.02	42.72 ± 2.45	46.88 ± 9.73	53.72 ± 5.67
Welch *t*	4.251	4.078	4.277	4.927	3.657	12.687	3.555	8.078
*p*	< 0.001	< 0.001	< 0.001	< 0.001	< 0.001	< 0.001	< 0.001	< 0.001
Hedges' *g* (95% CI)	0.659 (0.337– 0.982)	0.597 (0.276– 0.918)	0.658 (0.335– 0.980)	0.745 (0.421– 1.070)	0.573 (0.253– 0.894)	1.752 (1.384– 2.121)	0.569 (0.249– 0.889)	1.239 (0.896 – 1.581)

**TABLE 4:** Comparison of recorded cognitive task scores between the two groups (points).

Groups	Symbol Coding	CPT‐IP	Stroop Color‐Word Test	HVLT‐R	BVMT‐R	Mazes subtest	MSCEIT
Positive group (*n* = 93)	40.48 ± 4.11	12.37 ± 1.25	54.98 ± 3.17	20.14 ± 3.52	18.39 ± 3.17	14.29 ± 1.58	50.69 ± 4.77
Negative group (*n* = 67)	37.90 ± 3.51	11.62 ± 0.61	53.10 ± 2.52	17.79 ± 2.51	16.61 ± 2.87	12.12 ± 0.41	47.94 ± 4.86
Welch *t*	4.282	4.954	4.180	4.927	3.699	12.687	3.555
*p*	< 0.001	< 0.001	< 0.001	< 0.001	< 0.001	< 0.001	< 0.001
Hedges' *g* (95% CI)	0.665 (0.343– 0.988)	0.715 (0.391– 1.038)	0.642 (0.320– 0.964)	0.745 (0.421– 1.070)	0.580 (0.260– 0.901)	1.752 (1.384– 2.121)	0.569 (0.249– 0.889)

Abbreviations: BVMT‐R, Brief Visuospatial Memory Test‐Revised; CPT‐IP, Continuous Performance Test‐Identical Pairs; HVLT‐R, Hopkins Verbal Learning Test‐Revised; MSCEIT, Mayer‐Salovey‐Caruso Emotional Intelligence Test.

### 3.4 Correlation Analysis

Spearman rank correlation analysis showed that PANSS positive and negative symptom scores were inversely correlated (rho = −0.580, *p* < 0.001). PANSS negative symptom scores were inversely correlated with the recorded cognitive total score and PSP score (rho = −0.350 and −0.464, respectively; both *p* < 0.001). PANSS positive symptom scores were positively correlated with the recorded cognitive total score and PSP score (rho = 0.652 and 0.781, respectively; both *p* < 0.001). The recorded cognitive total score was positively correlated with PSP score (rho = 0.655, *p* < 0.001) (Table 5).

**TABLE 5:** Correlations of PANSS positive and negative symptom severity with cognitive and social functioning.

Variables		PANSS negative symptom score	PANSS positive symptom score	Recorded cognitive total score	PSP score
PANSS negative symptom score	rho	1.000	−0.580	−0.350	−0.464
	*p*	—	< 0.001	< 0.001	< 0.001
PANSS positive symptom score	rho	−0.580	1.000	0.652	0.781
	*p*	< 0.001	—	< 0.001	< 0.001
Recorded cognitive total score	rho	−0.350	0.652	1.000	0.655
	*p*	< 0.001	< 0.001	—	< 0.001
PSP score	rho	−0.464	0.781	0.655	1.000
	*p*	< 0.001	< 0.001	< 0.001	—

*Note*: Spearman's rho is the effect‐size measure for the correlation analyses. Statistical significance was determined from the corresponding two‐sided *p* value, not from an arbitrary threshold for the magnitude of the coefficient.

## 4 Discussion

Schizophrenia is a chronic and treatment‐resistant psychiatric disorder with an unclear etiology. In addition to positive symptoms such as hallucinations, delusions, and excitement, or negative symptoms such as affective blunting, impaired emotional communication, and apathy, patients commonly present with cognitive impairment, which is a prominent and characteristic feature of the disorder (Hui et al. 2025). Previous study has reported that approximately 75%85% of patients with schizophrenia exhibit varying degrees of cognitive decline (Horan et al. 2025). In a study by Sumei et al. (2025) involving 190 clinically stable patients with schizophrenia, 148 showed cognitive impairment, corresponding to a prevalence of 77.89%, with severe impairment accounting for approximately 26.32%. Analysis of schizophrenia by clinical symptom profiles has revealed differences in the extent of cognitive impairment. In the present study, patients were grouped according to PANSS positive and negative symptom scores. The results showed that the negative group had lower recorded cognitive total scores and lower scores across all seven recorded cognitive domains compared with the positive group. These findings indicate that patients with predominant negative symptoms experience broader and more severe cognitive impairment. This may be related to earlier and more insidious onset of negative symptoms. Moreover, numerous studies have shown that patients with negative symptoms often exhibit prefrontal cortex (PFC) dysfunction (Fuentes‐Claramonte et al. 2022; Xyangyi et al. 2026)^.^ The PFC is widely regarded as a hub of higher order cognitive function, playing critical roles in working memory, executive function, and attention. PFC hypofunction can therefore lead to deficits across multiple cognitive domains (Cai et al. 2025). Such hypofunction may be associated with dysregulation of neurotransmitter systems, including dopaminergic and glutamatergic pathways, resulting in neurotransmitter imbalance and subsequent cognitive deficits (K. X. Zhang, Yuan, et al., 2025). In the robust correlation analysis, PANSS negative symptom scores showed a modest inverse association with the recorded cognitive total score (Spearman rho = −0.350, *p* < 0.001).

The observed between‐group pattern is consistent with higher‐level evidence showing that negative symptoms are associated with multi‐domain cognitive impairment and that cognitive performance is related to functional outcomes (Au‐Yeung et al. 2023; Fett et al. 2011). The present findings extend this evidence by comparing symptom‐predominant clinical profiles within the same sample.

This study also found that PSP scores were lower in the negative‐symptom group compared with the positive‐symptom group, indicating more severe social functional impairment in patients with predominant negative symptoms. The underlying mechanism may be linked to cognitive deficits. For example, attention deficits can hinder patients from accurately focusing on verbal, facial, and gestural cues in social interactions, impairing their ability to interpret information and respond appropriately. Over time, this may lead to reduced social engagement and a contraction of the patient's interpersonal network (Cui et al. 2025; Li et al. 2025). Similarly, impaired working memory can make it difficult for patients to remember multiple tasks and deadlines in occupational settings, increasing the risk of work‐related errors and unemployment, which further compromises social function (Chen et al. 2025; Liu et al. 2025). Spearman analysis showed that PANSS negative symptom scores were inversely correlated with PSP score (rho = −0.464), while the recorded cognitive total score was positively correlated with PSP score (rho = 0.655; both *p* < 0.001). These associations support a relationship among symptom severity, cognition, and social functioning but do not establish a causal pathway. In addition, behaviors associated with negative symptoms, such as affective flattening, avolition, and social withdrawal, can directly hinder patients’ participation in social roles and activities, reducing motivation and capacity to engage socially, thereby weakening social support networks (Dong et al. 2025; Shi et al. 2024). Cognitive impairment further exacerbates social passivity and withdrawal, limiting functional recovery (Borkent et al. 2025). In the pooled sample, PANSS positive symptom scores were positively correlated with the recorded cognitive total score and PSP score (rho = 0.652 and 0.781, respectively; both *p* < 0.001). Because symptom scores were also used to define the predominant symptom groups, these pooled correlations should be interpreted descriptively and not as evidence that greater positive symptom severity improves cognition or functioning.

In summary, cognitive performance differs among patients with schizophrenia according to predominant clinical symptom profile. Patients in the negative‐symptom group showed poorer cognitive performance and social functioning than those in the positive‐symptom group. The cross‐sectional associations should be interpreted as descriptive and require confirmation in longitudinal studies.

## Author Contributions

**Xuezhen Jia**: conceptualization, methodology, project administration, resources, supervision, Writing – review and editing. **Kanghua Zhang**: writing – original draft, data curation, investigation, software, visualization. **Yu Mu**: writing – review and editing, formal analysis, validation. **Yao Chen**: conceptualization, data curation, formal analysis, investigation, methodology, writing – original draft.

## Funding

The authors have nothing to report.

## Ethics Statement

This study was approved by the Ethics Committee of Nanjing Pukou People's Hospital (Approval No. 2023‐SR‐025). The study was conducted in accordance with the Declaration of Helsinki. Due to the retrospective nature of the study, the requirement for informed consent was waived. All patient data were anonymized prior to analysis.

## Consent

The authors have nothing to report.

## Conflicts of Interest

The authors declare no conflicts of interest

## Data Availability

The data used and/or analyzed during the current study are available from the corresponding author.
